# Characterization of Coxsackievirus A6- and Enterovirus 71-Associated Hand Foot and Mouth Disease in Beijing, China, from 2013 to 2015

**DOI:** 10.3389/fmicb.2016.00391

**Published:** 2016-03-30

**Authors:** Jie Li, Ying Sun, Yiwei Du, Yuxiang Yan, Da Huo, Yuan Liu, Xiaoxia Peng, Yang Yang, Fen Liu, Changying Lin, Zhichao Liang, Lei Jia, Lijuan Chen, Quanyi Wang, Yan He

**Affiliations:** ^1^Department of Epidemiology and Biostatistics, School of Public Health, Capital Medical UniversityBeijing, China; ^2^Beijing Center for Disease Prevention and Control, Institute for Infectious Disease and Endemic Disease ControlBeijing, China

**Keywords:** hand foot and mouth disease, epidemic, Coxsackievirus A6, enterovirus 71, clinical features

## Abstract

**Background:** Etiology surveillance of Hand Foot and Mouth disease (HFMD) in Beijing showed that Coxsackievirus A6 (CVA6) became the major pathogen of HFMD in 2013 and 2015. In order to understand the epidemiological characteristics and clinical manifestations of CVA6-associated HFMD, a comparison study among CVA6-, EV71- (Enterovirus 71), and CVA16- (Coxsackievirus A16) associated HFMD was performed.

**Methods:** Epidemiological characteristics and clinical manifestations among CVA6-, EV71- and CVA16-associated mild or severe cases were compared from 2013 to 2015. VP1 gene of CVA6 and EV71 from mild cases, severe cases were sequenced, aligned, and compared with strains from 2009 to 2015 in Beijing and strains available in GenBank. Phylogenetic tree was constructed by neighbor-joining method.

**Results:** CVA6 became the predominant causative agent of HFMD and accounted for 35.4 and 36.9% of total positive cases in 2013 and 2015, respectively. From 2013 to 2015, a total of 305 severe cases and 7 fatal cases were reported. CVA6 and EV71 were responsible for 57.5% of the severe cases. Five out six samples from fatal cases were identified as EV71. High fever, onychomadesis, and decrustation were the typical symptoms of CVA6-associated mild HFMD. CVA6-associated severe cases were characterized by high fever with shorter duration and twitch compared with EV71-associated severe cases which were characterized by poor mental condition, abnormal pupil, and vomiting. Poor mental condition, lung wet rales, abnormal pupil, and tachycardia were the most common clinical features of fatal cases. The percentage of lymphocyte in CVA6-associated cases was significantly lower than that of EV71. High percentage of lymphocyte and low percentage of neutrophils were the typical characteristics of fatal cases. VP1 sequences between CVA6- or EV71-associated mild and severe cases were highly homologous.

**Conclusion:** CVA6 became one of the major pathogens of HFMD in 2013 and 2015 in Beijing. Epidemiological characteristics, clinical manifestations of CVA6-, EV71- and CVA16-associated cases in this study enriched the definition of HFMD caused by different pathogens and shed light to accurate diagnosis, appropriate treatment and effective prevention of HFMD.

## Introduction

Hand Foot and Mouth disease (HFMD) is a very common infectious disease usually associated with children younger than 5 years old. It is characterized by fever, sores or ulcers in the mouth and a rash with blisters. Until now there's still no effective treatment against this disease. In the past few decades, this disease has been reported all over the world, especially in the Asian-Pacific region (Pallansch Ma, 2001; Zhang et al., 2009; Xing et al., 2014).

Human enteroviruses (HEVs) belong to the genus Enterovirus, family Picornaviridae, and are divided into four species: HEV-A, HEV-B, HEV-C, and HEV-D on the basis of the phylogenetic properties of the viruses (Pöyry et al., 1996; Hyypiä et al., 1997). HFMD is most often caused by HEV-A species which now includes 18 serotypes (CVA2–8, CVA10, CVA12, CVA14, CVA16, EV71, EV76, EV 89–92, and EV114; Zhang et al., 2011). HEV-A species with CVA16 and EV71 are the two major serotypes agents of HFMD. CVA16 has contributed to the serious outbreaks of HFMD in China since 2008 (Xing et al., 2014) and has been endemic in Southeast Asia and the Pacific region for decades (Tu et al., 2007; Iwai et al., 2009). Illness caused by CVA16 infection is usually mild (Chang et al., 1999), whereas EV71, a neurotropic virus, is responsible for severe central nervous system complications (Chang et al., 1999). Large outbreaks caused by EV71 with high mortality and morbidity have been reported in Taiwan (Ho et al., 1999), Anhui (Zhang et al., 2010), Shandong (Zhang et al., 2009) in China, and some other countries as well (Gilbert et al., 1988; Cardosa et al., 1999; Ho et al., 1999; Chan et al., 2003).

In recent years, CVA6 was frequently associated with HFMD outbreaks in some countries such as Japan (Fujimoto et al., 2012), Singapore (Wu et al., 2010), Thailand (Puenpa et al., 2013), Finland (Osterback et al., 2010) and Spain (Guimbao et al., 2010; Haneke, 2010). In 2013, the etiology surveillance system in Beijing, as well as some other provinces or cities in China including Guangdong (Zeng et al., 2015), Tianjin (Tan et al., 2015), Nanjing (Hu et al., 2015), and Shenzhen (Li et al., 2014) found that positive rate of CVA6 increased dramatically and became one of the major pathogens of HFMD. Since then, CVA6 has spread in the mainland China and attracted considerable attention. However, few studies were performed to compare the epidemiology, clinical features among the different pathogen-associated HFMD in China, which were essential for deeply understanding the features of HFMD, and also be helpful for the diagnosis and treatment of the disease.

VP1 is the major surface-accessible protein in the mature enterovirus virion (Acharya et al., 1989) and contains a number of important neutralization epitopes (Minor, 1990). It has been proved to have an excellent genetic correlation with the EV serotype (Oberste et al., 1999a) and has been widely used for virus identification and evolutionary studies (Oberste et al., 1999b). In this study, we performed genetic analyses on VP1 sequences of CVA6 and EV71 from mild and severe cases to investigate if there's any linkage between the VP1 sequence and disease severity.

## Materials and methods

### Case definitions

A clinical case of HFMD was defined as oral ulcers, maculopapular or vesicular rash on the hands, feet and buttock, accompanied with or without fever. Patients were classified as severe if they had any neurological complications (aseptic meningitis, encephalitis, encephalomyelitis, acute flaccid paralysis, or autonomic nervous system dysregulation), or cardiopulmonary complications (pulmonary edema, pulmonary hemorrhage, or cardiorespiratory failure), or both (Xing et al., 2014).

### HFMD surveillance system in Beijing

In China, since 2008, clinicians and hospitals are required to report clinical cases of HFMD to National Notifiable Infectious Disease Surveillance System (NNIDSS) within 24 h of diagnosis (Xing et al., 2014). More than 400 hospitals located in Beijing were involved in the surveillance system. All the clinically diagnosed severe cases were transferred to two designated hospitals, Ditan Hospital and Youan Hospital to get treatment.

In order to get the information about the causative agents of HFMD, year-round etiology surveillance program was conducted as well. This etiology surveillance program was performed in 18 sentinel hospitals from 18 districts of Beijing. First five outpatients diagnosed as HFMD each month in 18 hospitals were included in this program. At each clinic, trained technician collected throat swab samples from these patients for serotyping with prior informed consent. All the specimens were sent to district CDC where EV, EV71, and CVA16 were identified by real-time-PCR. Specimens of non-EV71, non-CVA16 EV was sent to Beijing CDC for detail EV serotyping, virus isolating and VP1 gene sequencing.

### Participants, sample and data collection from mild HFMD cases

According to the geographical distribution of 18 districts in Beijing, 6 districts of Changping, Daxing, Fangshan, Haidian, Shunyi, and Tongzhou which are around the downtown of Beijing, were selected to participate in this study during July to December of 2013. The first 15 patients clinically diagnosed with HFMD infection each week were included in this study and were followed up two times for 9 weeks. Questionnaires were designed and certain personnel were trained to collect information of all infected cases through telephone counseling at the end of the first month and the last day of the ninth week.

### Participants, sample and data collection from severe and fatal HFMD cases

In order to get more information about the severe cases, we have attempted to collect the detailed information and specimens from all of the severe cases in Beijing from 2013 to 2015. With prior written informed consent, trained personnel filled out the questionnaires and collected specimens which would be sent to district CDC for agent identification. A comparison study of epidemiology and clinical manifestations between CVA6-, and EV71- associated severe HFMD cases was performed from 2013 to 2015.

### Samples processing mode, nucleotide extraction and virus identification

The specimens stored in MEM were vibrated violently for 1 min and centrifuged at 4000 g for 20 min prior to nucleotide extraction. Total nucleotide extraction was carried out with a Roche MagNA Pure LC 2.0 nucleic extraction system (ROCHE, Co, USA) using MagNA Pure LC Total Nucleic Acid Isolation Kit–Large Volume (ROCHE, Co, USA), according to the manufacturer's instructions. EV, EV71, and CVA16 were identified with real time RT-PCR Kit (DAAN Gene, Guangzhou, China). Serotype of non-EV71 non-CVA16 EV was identified by detecting the VP1 region of enterovirus positive samples according to the previously described method (Nix et al., 2006). Complete nucleotide sequences of VP1 genes were amplified using specific primers as previously described (Perera et al., 2007; Zhang et al., 2009; Sinclair et al., 2014). PCR products of complete VP1 genes were purified and sequenced using ABI PRISM310 Genetic Analyzer, respectively.

### Phylogenetic analysis of CVA6 and EV71 strains from mild and severe cases

In order to further investigate the homology of mild and severe strains isolated from EV71 or CVA6 infective patients, sequence analysis of the complete gene of VP1 was performed using the Molecular Evolutionary Genetics Analysis Version 6.0 (MEGA6). The phylogenetic tree was constructed by neighbor-joining method with bootstrapping of 1000 replicates. A total of 25 CVA6 isolates (21 isolates from mild cases and 4 from severe cases) and 27 EV71 isolates (19 isolates from mild cases, 7 from severe cases, and 1 from fatal case) which were representative in Beijing were aligned and compared with the corresponding region of CVA6 and EV71 isolates from 2009 to 2012 in Beijing and strains available at GenBank.

### Statistical analysis

In this study, we found that CVA6, CVA16, and EV71 were the major causative agents of mild HFMD and CVA6 and EV71 were responsible for major part of the severe cases in Beijing. In order to find out the typical features of mild cases and severe cases due to different pathogens, epidemiological information and clinical manifestations were compared.

Statistical analysis was conducted by SPSS17 software (IBM SPSS Inc., Chicago, IL, USA). Abnormal distribution continuous variables were described using median with 25th and 75th percentiles. Frequencies and proportions were used for categorical variables.

Nonparametric test (Kruskal–Wallis test) was used to compare the abnormal distribution continuous variables among three groups of CVA6-, CVA16- and EV71-associated mild cases. Pearson's χ^2^ test or Fisher's exact test was used to compare the difference of categorical variables among three groups of CVA6-, CVA16- and EV71-associated cases. If the comparison showed significant difference among three groups, Mann–Whitney *U*-test corrected by Bonferroni test will be performed.

Nonparametric test (Mann–Whitney *U*-test) was used to compare the difference of abnormal distribution continuous variables between two groups of CVA6- and EV71-associated severe cases. Pearson's χ^2^ test or Fisher's exact test was used to compare the difference of categorical variables between two groups of CVA6- and EV71-associated severe cases. A *p*-value of < 0.05 (2-sided significance testing) was considered statistically significant in above analyses.

### Ethics statement

This study was in compliance with the Helsinki Declaration and was approved by the Human Research Ethics Committee of Beijing CDC. Sample collection in this study was agreed by either the patient or the patient's guardian as appropriate with prior informed consent.

## Results

### CVA6 emerged as the major pathogen of HFMD in 2013 and 2015

The epidemiology and pathogen distribution of HFMD cases in Beijing from 2009 to 2015 was shown in Table 1. The annual number of reported HFMD cases ranged from 24,264 to 47,440, with the corresponding annual incidence rates ranged from 143.1 to 258.6 cases/100,000 populations. Patients less than 5 years of age accounted for more than 80% of all reported cases, the male cases were about 1.5 times of the female cases. The proportion of severe and fatal cases reached the peak of 1.3% and 0.4%0, respectively in 2010 (Table 1).

**Table 1:** **The epidemiology and pathogen distribution of HFMD cases in Beijing, China, 2009–2015**.

**Year**	**2009**	**2010**	**2011**	**2012**	**2013**	**2014**	**2015(Jan–Sep)**	***P*^a^**
Case Number	24264	45385	30843	38528	33144	47440	25111	NA
Population	16949994	17550006	1961257	20186012	20692994	21148000	NA	NA
Incidence rate (/10^5^)	143.1	258.6	157.3	190.9	160.2	224.3	NA	NA
Male (%)	14826 (61.1)	27372 (60.3)	18474 (59.9)	23079 (59.9)	20392 (61.5)	27992(59.0)	14951(59.5)	0.212
Age^a^	2.0 (1.0–3.0)	2.0 (1.0–4.0)	3.0 (1.0–4.0)	3.0 (1.0–4.0)	2.0 (1.0–4.0)	3.0 (2.0–4.0)	3.0 (2.0–4.0)	< 0.001
Fatal cases (%0)	4 (0.2)	18 (0.4)	5 (0.2)	4 (0.1)	2 (0.1)	2 (0.0)	1 (0.0)	0.005
Severe cases (%)	104 (0.4)	610 (1.3)	278 (0.9)	356 (0.9)	152 (0.5)	111(0.2)	42 (0.2)	< 0.001
Etiology examination (n)	1769	4174	3179	4791	2943	2570	1150	NA
EV Positive sample (n)	1204	2285	1508	2194	1762	1636	851	< 0.001
EV71 (%)	420 (34.9)	950 (41.6)	608 (40.3)	738 (33.6)	458 (26.0)	616 (37.7)	111 (13.0)	< 0.001
CVA16 (%)	634 (52.7)	716 (31.3)	666 (44.2)	1191 (54.3)	519 (29.5)	729 (44.6)	246 (28.9)	< 0.001
Other EV (%)	150 (12.5)	602 (26.3)	223 (14.8)	233 (10.6)	870	290	494	< 0.001
CVA6 (%)	NA	NA	NA	NA	623 (35.4)	50 (3.1)	314 (36.9)	NA
CVA10 (%)	NA	NA	NA	NA	5 (0.3)	22 (1.3)	3 (0.4)	NA

Age

a *, presented as median (p25, p75)*.

**Figure 1:**
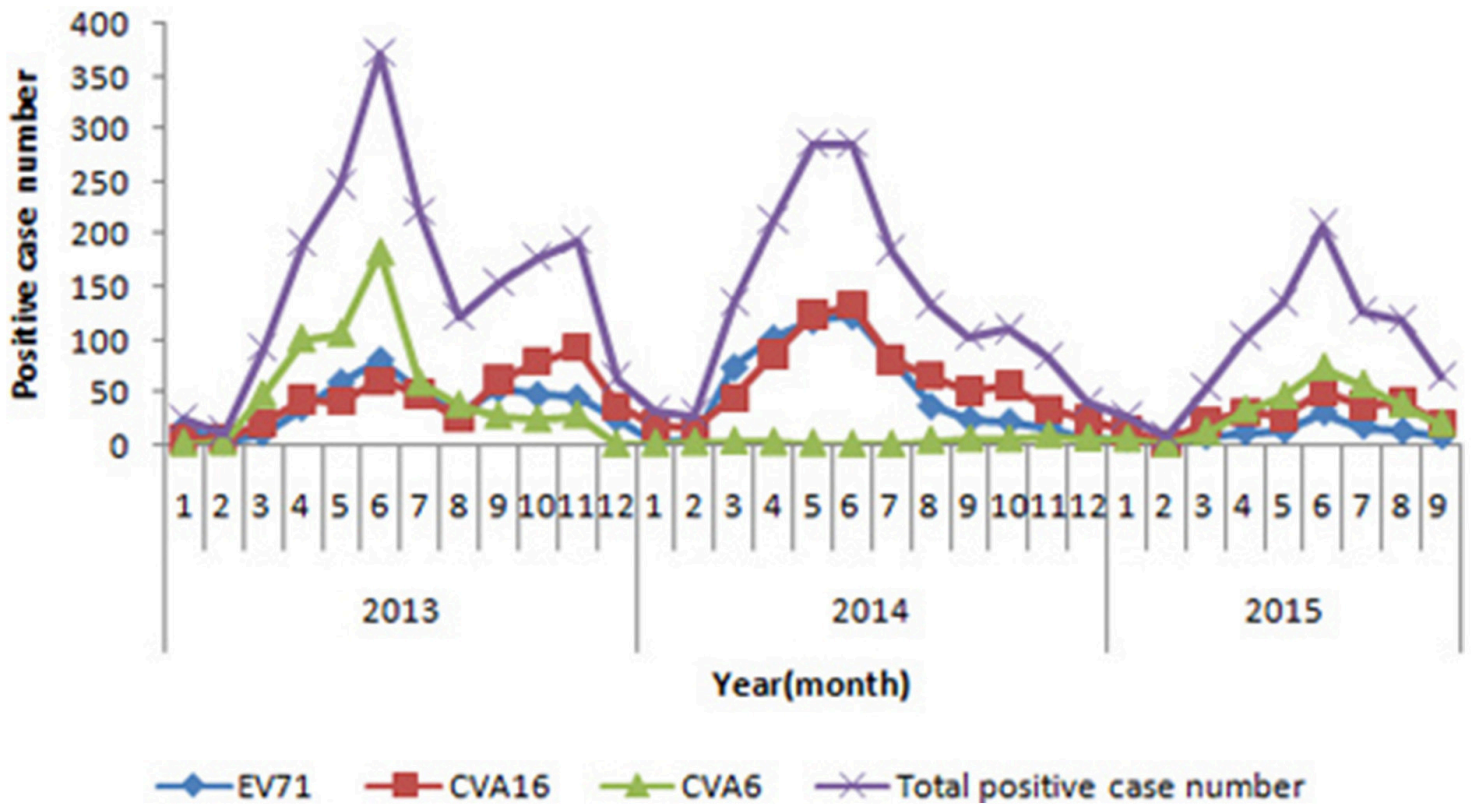
**Monthly distribution of enterovirus serotypes in laboratory-confirmed HFMD cases in Beijing, during 2013 and 2015**.

In 2009, 2011, 2012, and 2014, CVA16 was identified as the major pathogen of HFMD. In 2010, EV71 replaced CVA16 and became the most common pathogen associated with HFMD. While in 2013, CVA6 surpassed both CVA16 and EV71, became the predominant agent of HFMD and accounted for 35.4% of total positive cases. In 2014, CVA6-associated cases decreased and accounted for 3.1% of the positive cases. From January to September in 2015, CVA6 positive rate increased sharply again, overtook both CVA16 and EV71 and became the predominant causative agent of HFMD and accounted for 36.9% of positive cases (Figure 1).

### Epidemiology and clinical manifestations of CVA6-, EV71,- and CVA16-associated mild HFMD

From July through December of 2013, a total of 567 clinically diagnosed HFMD patients from six districts of Beijing were recruited and followed up 2 times for 9 weeks. All the patients participated in this study were diagnosed as mild cases and were successfully followed up. Among the patients, 62.4% (354/567) were male and the median age was 37 months. About 60.0% (340/567) cases were enterovirus positive and the proportion of CVA6, EV71, CVA16, and other serotype EV was 21.2 % (72/340), 21.2% (72/340), 42.9% (146/340), and 14.7% (50/340), respectively (Table 2).

**Table 2:** **The total number of samples from mild cases, severe cases, and fatal cases tested in this study**.

	**Mild cases (*n* = 567)**	**Severe cases (*n* = 270)**	**Fatal cases (*n* = 6)**
Negative case (%)	227 (40.0)	110 (40.7)	1 (16.7)
Positive case (%)	340 (60.0)	160 (59.3)	5 (83.3)
CVA6 (%)	72 (21.2)	41 (25.6)	0 (0.0)
EV71 (%)	72 (21.2)	51 (31.9)	5 (100.0)
CVA16 (%)	146 (42.9)	18 (11.3)	0 (0.0)
Other EV (%)	50 (14.7)	50 (31.3)	0 (0.0)

In order to get more detail information about the difference among CVA6-, CVA16,- and EV71-associated mild cases, epidemiological data and clinical features were compared and the results were shown in Table 3. Both the proportion of cases with symptom of fever and the proportion of cases with symptom of fever over 39°C among CVA6-associated cases were significantly higher than CVA16-(*P* < 0.001) and EV71-(*P* < 0.001) associated cases. Desquamation on the palms and soles after infection episode in CVA6-associated cases was significant higher than that in the CVA16- associated cases (*P* = 0.016). Proportion of cases with onychomadesis in CVA6-associated cases was significantly higher than CVA16-(*P* < 0.001) and EV71- (*P* < 0.001). All the patients with onychomadesis denied a past history of major systemic disease or nail trauma in the 8 weeks prior to the onset of the nail abnormalities.

**Table 3:** **Epidemiology and clinical features of CVA6-, EV71,- and CVA16-associated mild cases in Beijing, 2013**.

**Characteristics**	**CVA6 (*n* = 72)**	**CVA16 (*n* = 146)**	**EV71 (*n* = 72)**	***P***	***P*^a^**		
					**CVA6 vs. CVA16**	**CVA6 vs. EV71**	**CVA16 vs. EV71**
Age (month)^b^	35.5(18.5–53.5)	40.0(27.75–53.25)	34.0(17.0–51.5)	0.113	NA	NA	NA
**SEX**							
Male (%)	41(56.9)	96(65.8)	51(70.8)	0.206	NA	NA	NA
**OCCUPATION**							
Scattered children (%)	35(49.3)	54(37.0)	41(56.9)	0.053	NA	NA	NA
Children in Kindgarden (%)	29(40.8)	80(54.8)	26(36.1)				
Primary school (%)	7(9.9)	12(8.2)	5(6.9)				
^b^Median duration from onset to initial diagnosis	1(1.0–2.0)	1(0.0–1.0)	1(0.0–1.0)	**0.010**	**0.011**	**0.006**	0.475
^b^Rash duration	2(1.0–3.0)	2(1.0–2.0)	1(1.0–2.0)	**0.002**	0.030	<**0.001**	0.064
**RASH %**							
Hand	69(95.8)	141(96.6)	71(98.6)	0.621	NA	NA	NA
Foot	62(86.1)	112(76.7)	59(81.9)	0.240	NA	NA	NA
Mouth	58(80.6)	107(73.8)	50(69.4)	0.309	NA	NA	NA
Buttock	26(36.1)	37(25.5)	23(31.9)	0.241	NA	NA	NA
Vesicle	6(8.3)	12(8.2)	3(4.2)	0.509	NA	NA	NA
Pigmentation	9(12.5)	7(4.8)	3(4.2)	0.062	NA	NA	NA
Fever	53(73.6)	41(28.1)	26(36.1)	<**0.001**	<**0.001**	<**0.001**	0.227
Fever (≥39°C)	23(31.9)	11(7.5)	5(6.9)	<**0.001**	<**0.001**	<**0.001**	0.875
Desquamation	28(38.9)	34(23.3)	16(22.2)	**0.030**	**0.016**	0.030	0.860
Onychomadesis	13(18.1)	0(0.00)	1(1.4)	<**0.001**	<**0.001**	**0.001**	0.153

P

a *< 0.05/3 was considered to be significant*.

b *presented as median (p25, p75)*.

*P, from Pearson's χ^2^, Fisher's exact test or Rank test*.

*NA, data not available*.

*The bold values means significant difference was obtained between or among compared groups*.

### Epidemiology and clinical manifestations of CVA6- and EV71-associated severe HFMD

A total of 305 clinically diagnosed severe HFMD cases were reported from 2013 to the October in 2015. Information of 281 severe cases, which included 197 male and 84 female were collected with the median age of 23 months. About 63.7% (179/281) cases were initially diagnosed as HFMD. The most common clinical manifestations of severe cases were fever (100%) and rash (100%), followed by poor mental condition (50.9%), hyperarousal (39.5%), tremble of hand and foot (36.3%), twitch (31.3%), and vomiting (31.0%).

From 2013 to 2015, the throat swab samples from the 270 severe cases were collected and analyzed by real-time-PCR and 59.3% (160/270) cases were positive for enterovirus. The positive proportion of CVA6, EV71, CVA16, and other serotype EV were 25.6% (41/160), 31.9% (51/160), 11.3% (18/160), and 31.3% (50/160), respectively (Table 2).

Characteristics of 33 CVA6- and 46 EV71-associated severe cases with complete information were compared and the result was showed in Table 4. Twenty-two (66.7%) of CVA6-associated severe cases and thirty-two (69.6%) of EV71-associated cases were male (*P* = 0.785) with the median ages of 22 and 23 months (*P* = 0.505), respectively. The median duration from disease onset to diagnosis as severe case among CVA6–associated severe cases were significantly shorter than that of EV71-associated severe cases (*P* = 0.002).

**Table 4:** **Epidemiology and clinical features of CVA6- and EV71- associated severe HFMD cases**.

**Characteristics**	**Severe cases (*n* = 281)**	**Fatal cases (*n* = 7)**	**Severe cases due to CVA6 or EV71**		
			**CVA6(*n* = 33)**	**EV71(*n* = 46)**	***P*^a^**
^b^Age (month)	23(17.0–41.0)	17.0(12.0–25.0)	22(17.5–37.5)	23(15.8–44.3)	0.505
**SEX (%)**					
Male	197(70.1)	5(71.4)	22(66.7)	32(69.6)	0.785
**OCCUPATION (%)**					
Scattered children	194(69.0)	6(85.7)	26(78.8)	29(63.0)	0.133
Children in Kindgarden	86(30.6)	1(14.3)	7(21.2)	17(37.0)	
Primary school	1(0.4)	0(0.0)	0(0.0)	0(0.0)	
^b^Median duration from onset to diagnosis	0(0.0–1.0)	1.0(0.0–2.0)	0(0.0–1.0)	1(0.0–2.0)	**0.031**
Diagnosed as HFMD at the initial visit (%)	179(63.7)	4(57.1)	20(60.6)	30(65.2)	0.675
^b^Median duration from onset to diagnosis of severe or death case	2(1.0–3.0)	2(2.0–3.0)	1(1.0–2.0)	3(2.0–3.0)	**0.002**
^b^Fever peak before visiting hospital (°C)	39(38.7–39.6)	38.9(38.5–39.3)	39.6(39–40)	39.0(38.6–39.3)	**0.001**
^b^Fever peak of the whole course (°C)	39.2(38.9–39.7)	39.0(38.7–39.3)	39.8(39.2–40.0)	39.1(39.0–39.6)	**0.007**
^b^Fever duration (day)	2(2.0–3.0)	3(3.0–4.0)	2(2.0–3.0)	3(2.0–4.0)	**0.009**
^b^Rash duration (day)	3(2.0–4.0)	3(2.5–4.5)	3(2.0–4.0)	3(2.3–4.0)	0.252
**RASH (%)**					
Hand	223(79.4)	5(71.4)	24(72.7)	42(91.3)	**0.028**
Foot	205(73.0)	5(71.4)	21(63.6)	41(89.1)	**0.007**
Mouth	218(77.6)	3(42.9)	30(90.9)	36(78.3)	0.135
Buttock	136(48.4)	2(28.6)	15(45.5)	25(54.3)	0.436
**SYMPTOM OF NERVOUS SYSTEM (%)**					
Headache	41(14.6)	0(0.0)	5(15.2)	8(17.4)	0.791
Poor Mental condition	143 (50.9)	6(85.7)	13(39.4)	29(63.0)	**0.038**
Hyperarousal	111 (39.5)	2(28.6)	17(51.5)	26(56.5)	0.659
Dysphoria	26 (9.3)	3(42.9)	4(12.1)	7(15.2)	0.754
Twitch	88 (31.3)	2(28.6)	17(51.5)	13(28.3)	**0.036**
Seizures	26 (9.3)	2(28.6)	3(9.1)	3(6.5)	0.671
Tremble of hand and foot	102 (36.3)	2(28.6)	16(48.5)	27(58.7)	0.369
Limb weakness	14 (5.0)	2(28.6)	3(9.1)	2(4.3)	0.644
Neck resistance	4(1.4)	0(0.0)	0(0.0)	1(2.2)	1.000
Abnormal tendon reflexes	6(2.1)	2(28.6)	0(0.0)	4(8.7)	0.136
Drowsiness	29 (10.3)	1(14.3)	2(6.1)	7(15.2)	0.291
Lethargy	7(2.5)	0(0.0)	0(0.0)	3(6.5)	0.261
Coma	5(1.8)	2(28.6)	0(0.0)	2(4.3)	0.507
Abnormal pupil	13(4.6)	4(57.1)	0(0.0)	7(15.2)	**0.038**
Vomiting	87 (31.0)	3(42.9)	7(21.2)	23(50)	**0.009**
**SYMPTOM OF RESPIRATORY SYSTEM (%)**					
Cough	44 (15.7)	2(28.6)	4(12.1)	8(23.9)	0.520
Ecphysesis	13 (4.6)	3(42.9)	1(3.0)	6(13.0)	0.229
Dyspnea	7(2.5)	2(28.6)	0(0.0)	4(8.7)	0.136
Lung wet rales	9(3.2)	5(71.4)	0(0.0)	4(8.7)	0.136
**SYMPTOM OF CIRCULATORY SYSTEM (%)**					
Cyanose of fingers, toes or lips	15 (5.3)	1(14.3)	2(6.1)	2(4.3)	1.000
Pale face and extremities	7(2.5)	3(42.9)	1(3.0)	3(6.5)	0.636
Pale body	3(1.1)	1(14.3)	0(0.0)	3(6.5)	0.261
Tachycardia	59 (21.0)	4(57.1)	7(21.2)	13(28.3)	0.477
Coolness of extremities	15(5.3)	2(28.6)	2(6.1)	4(8.7)	1.000
**LABORATORY TEST**					
WBC(10^9^/L)	11.9(9.5–15.1)	20.2(11.7–31.0)	12.8(11.1–16.4)	11.8(9.1–14.7)	0.132
Lymphocyte (%)	25.1(17.2–35.8)	72.6(61.4–77.4)	20.1(13.9–30.7)	28.5(22.0–37.9)	**0.004**
Neutrophil (%)	64.8(51.8–74.4)	22.4(15.4–31.1)	66.8(52.3–76.1)	64.9(51.9–72.2)	0.490

P

a *, from Chi-square test, Fisher's exact test or Mann–Whitney U-test corrected by Bonferroni test*.

b *presented as median (p25, p75)*.

*The bold values means significant difference was obtained between or among compared groups*.

The fever peak of the whole disease course of CVA6-associated severe case was significantly higher than that of EV71-associated cases (*P* = 0.007) and the median duration of fever among CVA6-associated cases was significantly shorter than that of EV71-associated cases (*P* = 0.007). Furthermore, the incidence of symptoms of poor mental condition, abnormal pupil and vomiting among CVA6-associated cases was significantly lower than that of EV71-associated cases (*P* = 0.038, *P* = 0.038, *P* = 0.009), respectively. While the incidence of twitch among CVA6-associated cases was significantly higher than that of EV71-associated cases (*P* = 0.036). Additionally, Lymphocyte percentage of CVA6 infected severe cases was significantly lower than that of EV71 infected severe cases (*P* = 0.004).

### Epidemiology and clinical manifestations of fatal HFMD cases

From 2013 to 2015, a total of 7 fatal cases which included 5 permanent residents and 2 migrants were reported (Table 3). Among the 7 fatal cases, 5 were male and the median age was 17.5 months. Five out of seven cases died in 3 days after HFMD onset. A total of 6 samples were collected from fatal cases and 5 samples were identified as EV71 positive. The most common clinical manifestations of fatal cases were fever (100%) and rash (100%), followed by poor mental condition (85.7%), lung wet rales (71.4%), abnormal pupil (57.1%), tachycardia (57.1%), dysphoria (42.9%), vomiting (42.9%), ecphysesis (42.9%), and pale face and extremities (42.9%). The median WBC counts, median lymphocyte percentage and median neutrophil percentage of fatal cases were 20.2^*^10^9^/L, 72.6 and 22.4%, respectively. WBC counts and lymphocyte percentage of all fatal cases were above the upper limit of the normal range, while neutrophil percentage of all fatal cases were lower than lower limit of the normal range.

### Phylogenetic analysis

**Figure 2:**
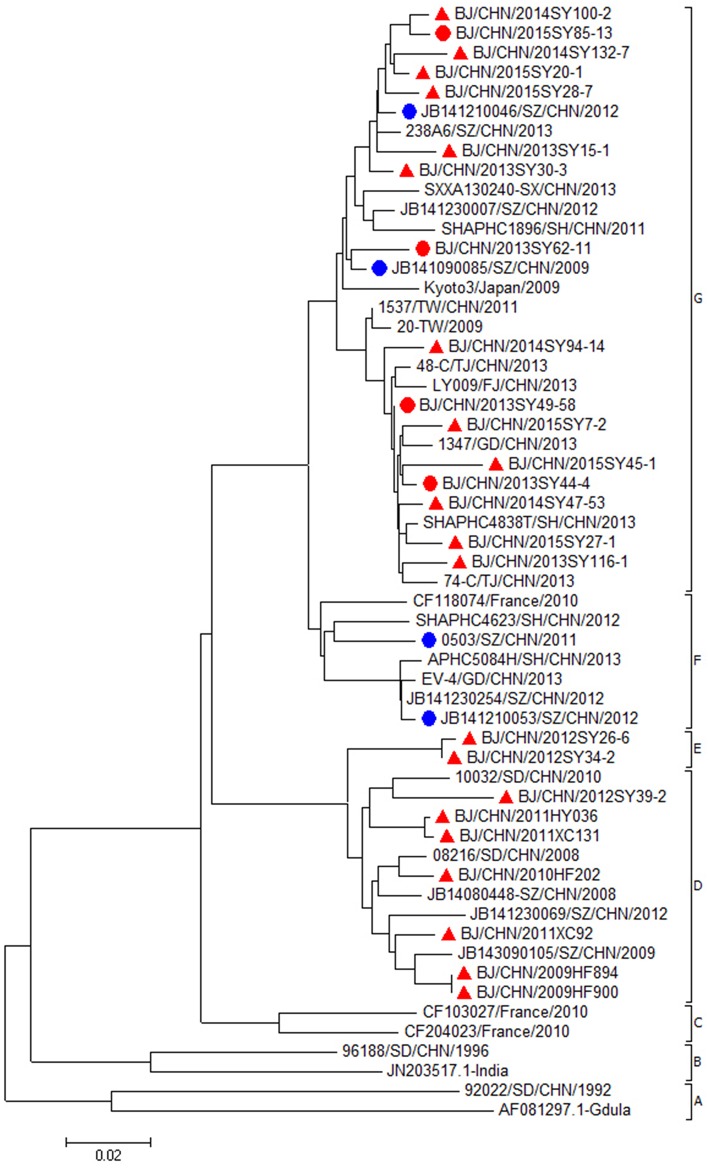
**Phylogenetic dendrogram based on the alignment of the complete VP1 gene sequence of CVA6 (915bp)**. CVA6 strains isolated from mild cases, severe cases in Beijing were indicated by a red triangle, a red dot, respectively. CVA6 strains isolated from severe cases in other cities were indicated by a blue dot.

All the CVA6 strains were classified into seven major clades (A–G) (Figure 2). CVA6 isolates in this study from 2013 to 2015 (20 strains from mild cases and 4 strains from severe cases) were all located in cluster G. The nucleotide and amino acid identities of the VP1 gene among mild CVA6 strains were 94.1–98.3% and 96.8–100.0%. The nucleotide and amino acid identities of the VP1 gene among severe CVA6 strains were 95.7–99.4% and 98.9–100.0%. CVA6 strains from mild cases shared nucleotide identity of 94.5–99.4% and amino acid identities of 97.8–99.5% with the strains from severe cases. Compared with CVA6 strains from severe cases in Shenzhen from 2009 to 2012, CVA6 isolates from severe cases in Beijing shared a nucleotide and amino acid identity of 94.5–98.5 and 97.8–99.6%.

**Figure 3:**
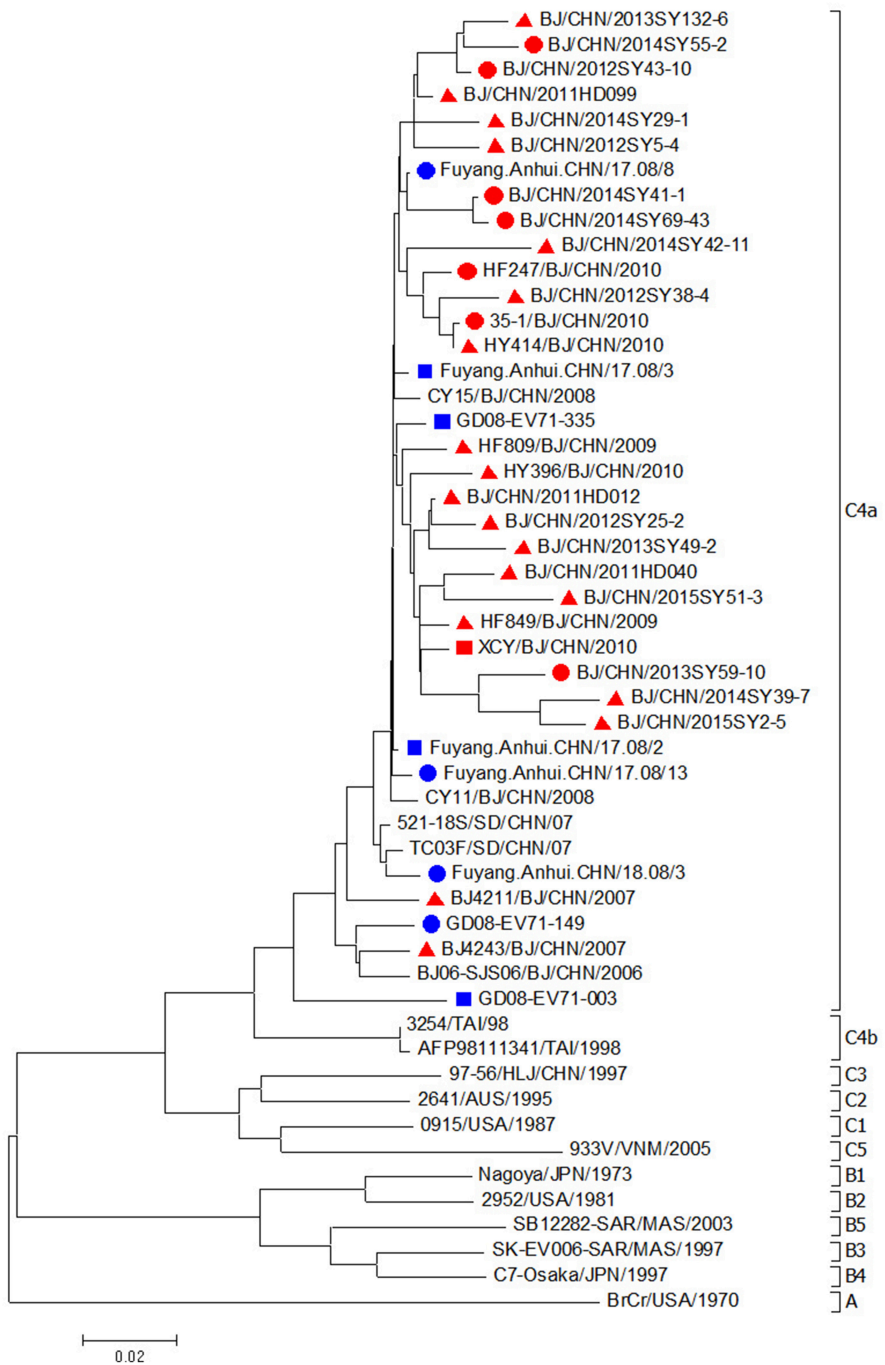
**Phylogenetic dendrogram based on the alignment of the complete VP1 gene sequence of EV71 (891bp)**. EV71 strains isolated from mild cases, severe cases and fatal cases in Beijing were indicated by a red triangle, a red dot and a red square, respectively. EV71 strains isolated from severe cases and fatal cases in other cities were indicated by a blue dot and a blue square, respectively.

EV71 isolates in this study from 2007 to 2015 (19 strains from mild cases, 7 strains from severe cases, and 1 strain from fatal case) were all located in the cluster of C4a genotype (Figure 3). The nucleotide and amino acid identities of the VP1 gene among mild EV71 strains were 93.3–99.9 and 97.5–100.0%. The nucleotide and amino acid identities of the VP1 gene among severe and fatal EV71 strains were 94.7–99.5 and 98.6–100.0%. EV71 strains from mild cases shared nucleotide identity of 93.3–98.9% and amino acid identities of 97.9–100.0% with the strains from severe and fatal cases. Compared with EV71 strains from severe cases or fatal cases in Anhui and Shandong, EV71 isolates from severe and fatal cases in Beijing shared a nucleotide identity of 92.4–98.7%, with the corresponding amino acid identity of about 98.6–100.0%.

## Discussion

In the past decade, numerous epidemics of HFMD have occurred in the Asia-Pacific region, which were mainly caused by EV71 and CVA16. Since 2013, the positive proportion of CVA6 has increased greatly and became the predominant pathogen of HFMD in several provinces of China (Hongyan et al., 2014; Lu et al., 2014; Tan et al., 2015; Zeng et al., 2015). The etiology surveillance on thousands of HFMD cases each year from 2009 to 2012 in Beijing showed that the positive rates of non-CVA16 and non-EV71 EV-associated cases ranged from 10.6 to 26.3%. The present study showed that CVA6 alone accounted for 35.4% (2013) and 36.9% (2015) of the total positive cases and surpassed CVA16 and EV71 became the predominant strain in Beijing. This observation suggested that non-CVA16, non-EV71 EV-associated HFMD should be monitored closely in order to give early warning when new serotypes emerge so as to downsize the prevalence and reduce the infected cases.

It has been reported that high fever (Lo et al., 2011), onychomadesis (Osterback et al., 2010; Wei et al., 2011; Fujimoto et al., 2012), pigmentation (Wei et al., 2011; Huang et al., 2013), vesicular rash (Ben-Chetrit et al., 2014; Feder et al., 2014; Hu et al., 2015), and desquamation (Feder et al., 2014) are associated with the CVA6 infected patients. In this study, results indicated that high fever, desquamation and onychomadesis were characterized manifestations of CVA6-associated cases. Pediatricians should be alert for the above characterized clinical presentations so that appropriate treatment measures could be provided. On the other hand, the above typical features of CVA6-associated mild cases might enrich the definition of HFMD which is very important for the disease prevention and control and provide useful information for the further study in the burden of disease.

In our study, we found that the total number of severe cases of HFMD in Beijing had an obviously drop from 2010 to 2015 which was in agreement with result of previous study (Huang et al., 2015). EV71 was still the leading serotype responsible for severe HFMD cases, especially fatal HFMD cases. While with the CVA6 becoming the major pathogen of HFMD, it accounted for an increase of severe cases in 2013 and 2015. Similar to the mild cases, the fever peak of CVA6-associated severe cases was significantly higher than that of EV71 infected cases which was accordant with previously reported studies (Hongyan et al., 2014; Yang et al., 2014). Based on the result we suspected that hyperpyrexia which was the typical clinical manifestation of CVA6 might have driven the parents or guardians to take their children to the hospital. As a result, patient's visiting to hospital and diagnosis of severe cases among CVA6 patients were significantly earlier than EV71 which might be a protective factor of a better prognosis. It has been widely known that EV71 is in its neurotropism to cause neurological complications (Wang and Liu, 2014). In this study we found that poor mental condition, abnormal pupil, and vomiting were the typical symptoms of EV71-associated severe cases compared with CVA6-associated severe cases. Besides fever and rash, poor mental condition, lung wet rales, abnormal pupil, and tachycardia were the most common clinical features of fatal cases. In this study, we observed about 36.3% severe cases and 42.9% fatal cases were misdiagnosed as the other disease at the initial visit to the hospital. According to a previous study (Pan et al., 2012), unable to be diagnosed as HFMD in the first visit to the hospital was an important risk factor of severe HFMD. Based on the above results we believed that a clear understanding of the disease might improve the diagnostic accuracy and therefore, appropriate treatment can be performed. On the other hand, the disease course of severe or fatal case was a continuous and dynamic procedure. It may be a reversible disease if the clinical symptoms were recognized in the early stage. Considering the rapid progression of this disease in which 5 out of 7 severe cases died in the first 3 days, we proposed that typical features were valuable for the clinician making a diagnosis as well as evaluating the stage of disease progression and thereby preventive measures could be instituted and effective treatment could be performed.

The response of the immune system of enterovirus infected cases was also evaluated. Our study showed that lymphocyte percentage of some CVA6- or EV71-associated severe cases were below the lower limit of normal range. This phenomenon was accordant with the result of Wang's study (Wang et al., 2003) in which the absolute lymphocyte count decreased dramatically at first and returned in several days. However, the lymphocyte percentage of all the fatal cases was much higher than the upper limit of normal range and the neutrophil percentage of all the fatal cases was lower than the lower limit of normal range. Based on this result, we suggested that dramatically increased lymphocyte percentage and decreased neutrophil percentage might be a sign of worsen condition. This could be used as a functional marker for EV71-related cases and potential treatment options.

Furthermore, in our study, EV71 was responsible for 5 out of 6 fatal cases. This result suggested that although the proportion of EV71 declined in recent years, considering that EV71 was prone to cause severe cases, even death, strategy of how to prevent the spreading of EV71 should still be paid attention to. Nowadays, many studies on EV71 vaccine are ongoing and three vaccines had completed their clinical trial (Zhu et al., 2013, 2014; Li et al., 2015). Previous clinical trials studies showed that EV71 vaccines were efficacy and safety, but did not protect the population from other serotypes infection (Chou et al., 2012; Chong et al., 2015). Considering the great change in the HFMD epidemic pattern, we suggested that the trend of HFMD epidemic should be monitored closely and the vaccine strategies against HFMD should be formulated based on the epidemic situation of HFMD.

In this study, both strains from mild and severe cases of CVA6 or EV71 were all located on the same cluster of the phylogenetic tree, indicating that they shared the single ancestor and were undistinguishable only depending on the VP1 sequence. According to a previous study, VP1-145E variants were mainly responsible for the development of viremia and neuropathogenesis and involving in the vivo fitness, and pathogenesis in EV71-infected individuals (Chang et al., 2012). In our study, we didn't find a link between the 145th amino acid of VP1 and disease severity, since 145E was a predominant form in both mild and severe cases. We suggested that severe cases couldn't be distinguished from mild cases only depending on the amino acid on this site. From our point, the immune response status of the host might play essential role in the whole course as well. Till now, there is no confirmative evidence about what kind of strains are prone to cause severe even fatal cases and what kind of person are easy to develop to severe case. Hence, more information, such as the genome of pathogen and the dynamic immunity status of host are needed to carry out further study.

In conclusion, CVA6 became the predominant causative agent of HFMD in Beijing, China, in 2013 and 2015. CVA6 and EV71 accounted for the majority of severe HFMD. Non-CVA16, non-EV71 EV-associated HFMD should be monitored closely in order to give early warning when new serotypes emerge so as to downsize the prevalence and reduce the infected cases. Typical clinical features enriched the definition about CVA6- and EV71-associated HFMD and were helpful for the diagnosis and treatment of the disease. EV71 was responsible for most of the fatal cases. Vaccine strategy against EV71 should be formulated base on the current epidemic situation of HFMD.

## Author contributions

JL carried out the experimental design, participated in the experiment and drafted the manuscript. YS and DH participated in the experimental design, performed the data analysis, and reviewed the manuscript. YY, XP, FL, and LJ participated in the clinical data collection, analysis, and reviewed the manuscript, YD, YL, YY, CL, ZL, and LC participated in the experiment and reviewed the manuscript. QW and YH contributed to the experimental design and coordination with District Centers for Disease Control and Prevention and provided a final review of this manuscript. All authors read and approved the final manuscript.

## Funding

This work was supported by grants of key projects in the National Science & Technology Pillar Program (2015BAI09B01). This work was also supported by the grant from Beijing Natural Science Foundation to YH (7132027) and the grant from Beijing Natural Science Foundation to JL (7164240).

### Conflict of interest statement

The authors declare that the research was conducted in the absence of any commercial or financial relationships that could be construed as a potential conflict of interest.

## Abbreviations

HFMD: Hand Foot and Mouth disease
CVA6: Coxsackievirus A6
CVA16: Coxsackievirus A16
EV71: Enterovirus 71
NNIDSS: National Notifiable Infectious Disease Surveillance System
MEGA6.06: Molecular Evolutionary Genetics Analysis Version 6.06.
